# Supplementary figures and images for: The protein segregase VCP/p97 promotes host antifungal defense via regulation of SYK activation
Source: PLoS Pathog. 2024 Oct 29;20(10):e1012674. doi: 10.1371/journal.ppat.1012674 (PMC11548748; doi:10.1371/journal.ppat.1012674)

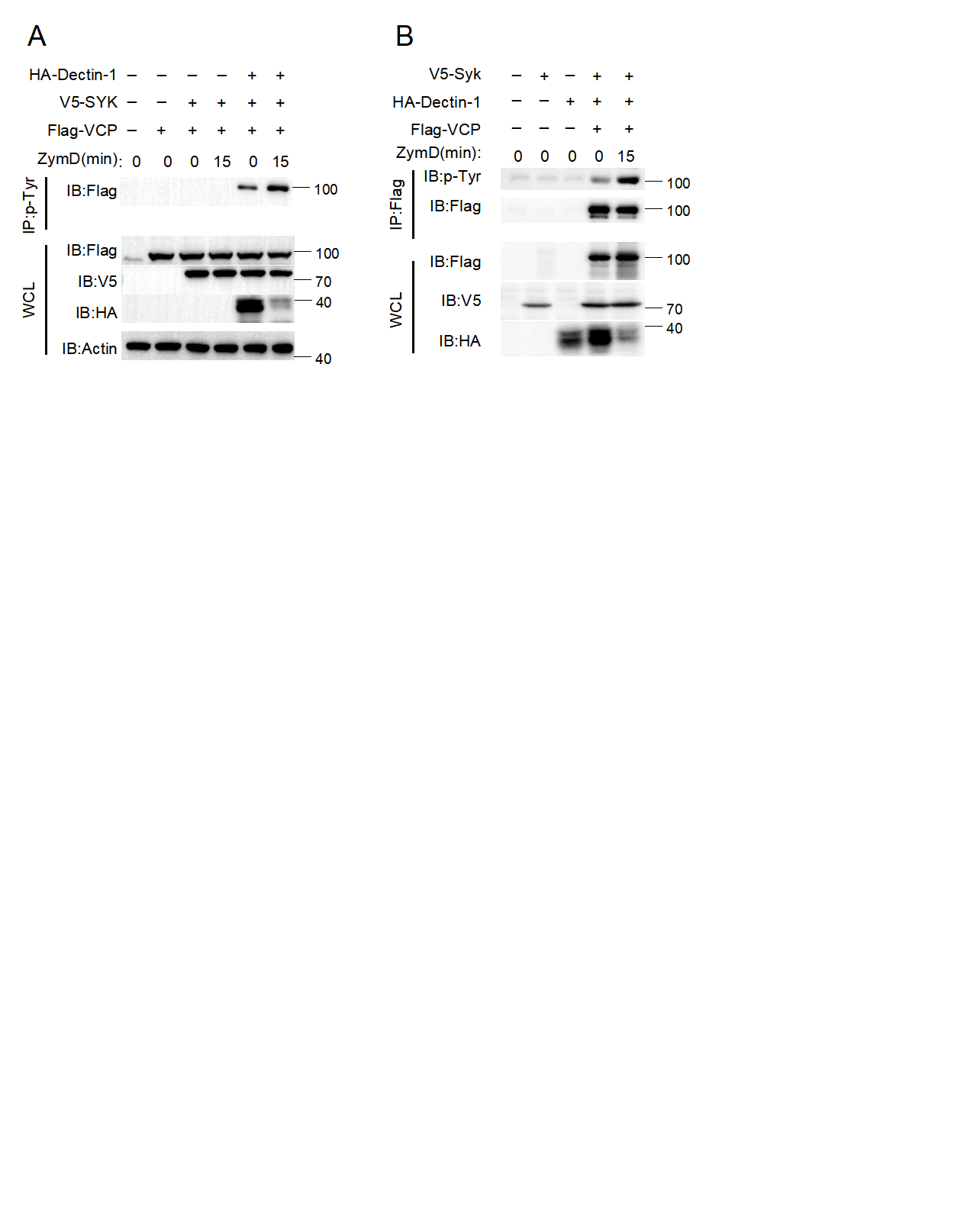

Supplement: S1 Fig — (A) HEK293T cells were transfected with the plasmids expressing Dectin-1, SYK, empty control or VCP plasmids for 36 h and then stimulated with ZymD (100 μg/ml) for indicated time. Cell lysates were subjected to IP with anti-pTyr and then immunoblotted with indicated antibodies. (B) HEK293T cells were transfected with the plasmids expressing Dectin-1, SYK, empty control or VCP plasmids for 36 h and then stimulated with ZymD (100 μg/ml) for indicated time. Cell lysates were subjected to IP with anti-Flag and then immunoblotted with indicated antibodies. In A-B, one representative experiment of three independent experiments is shown. (TIF) [file ppat.1012674.s001.tif]

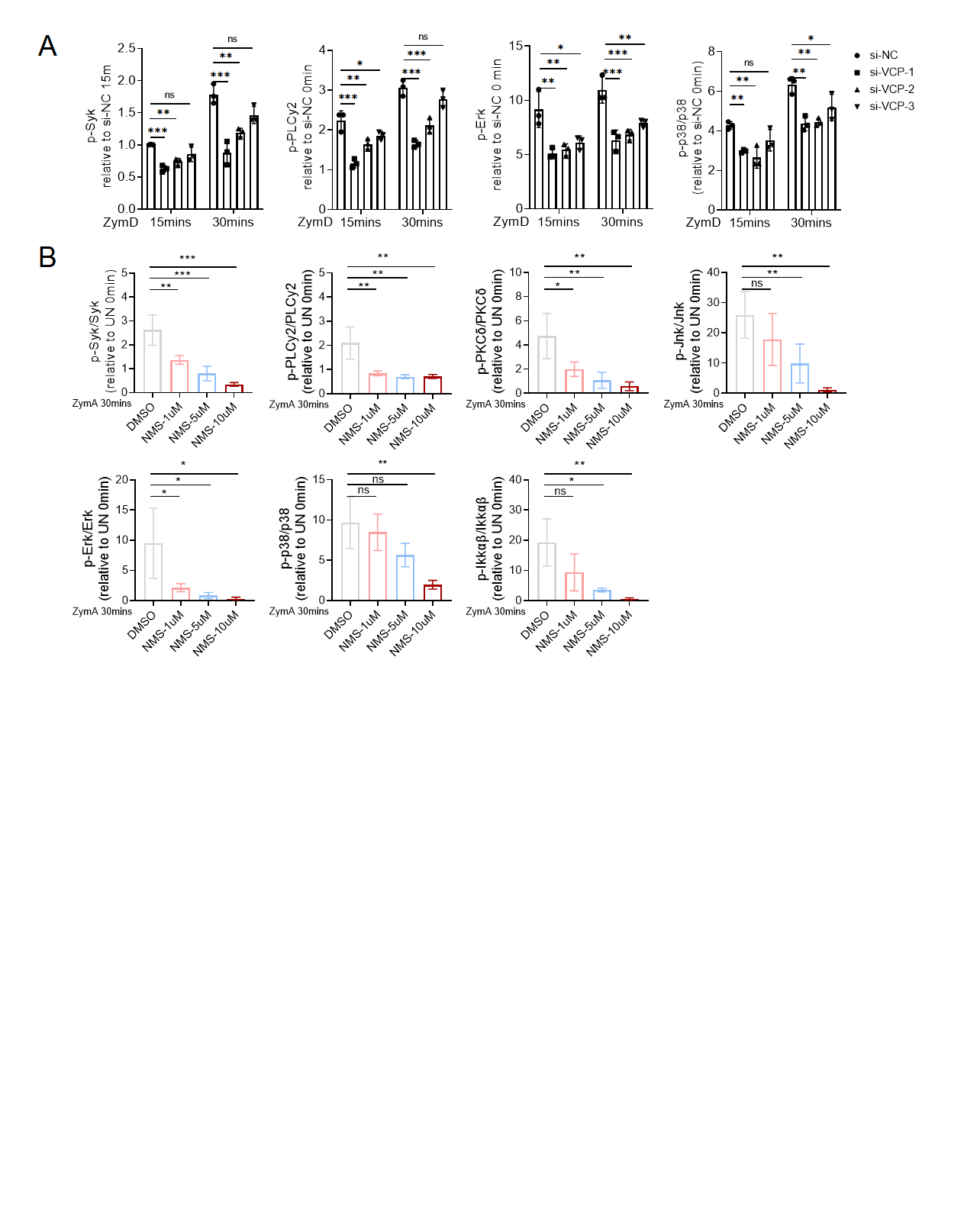

Supplement: S2 Fig — (A) Densitometric quantification of p-Syk, p-PLCγ2, p-Erk, and p-p38 from three independent experiments, as depicted in Fig 1E, was conducted using ImageJ. (B) Densitometric quantification of p-Syk, p-PLCγ2, p-PKCδ, p-Jnk, p-Erk, p-p38 and p-IKKα/β from three independent experiments, as shown in Fig 2B, was performed using ImageJ. Data are shown as mean ± SD and were analyzed by one-way ANOVA (A and B). (*: p<0.05; **: p<0.01; ***: p<0.001; ****: p<0.0001, ns: no significance). (TIF) [file ppat.1012674.s002.tif]

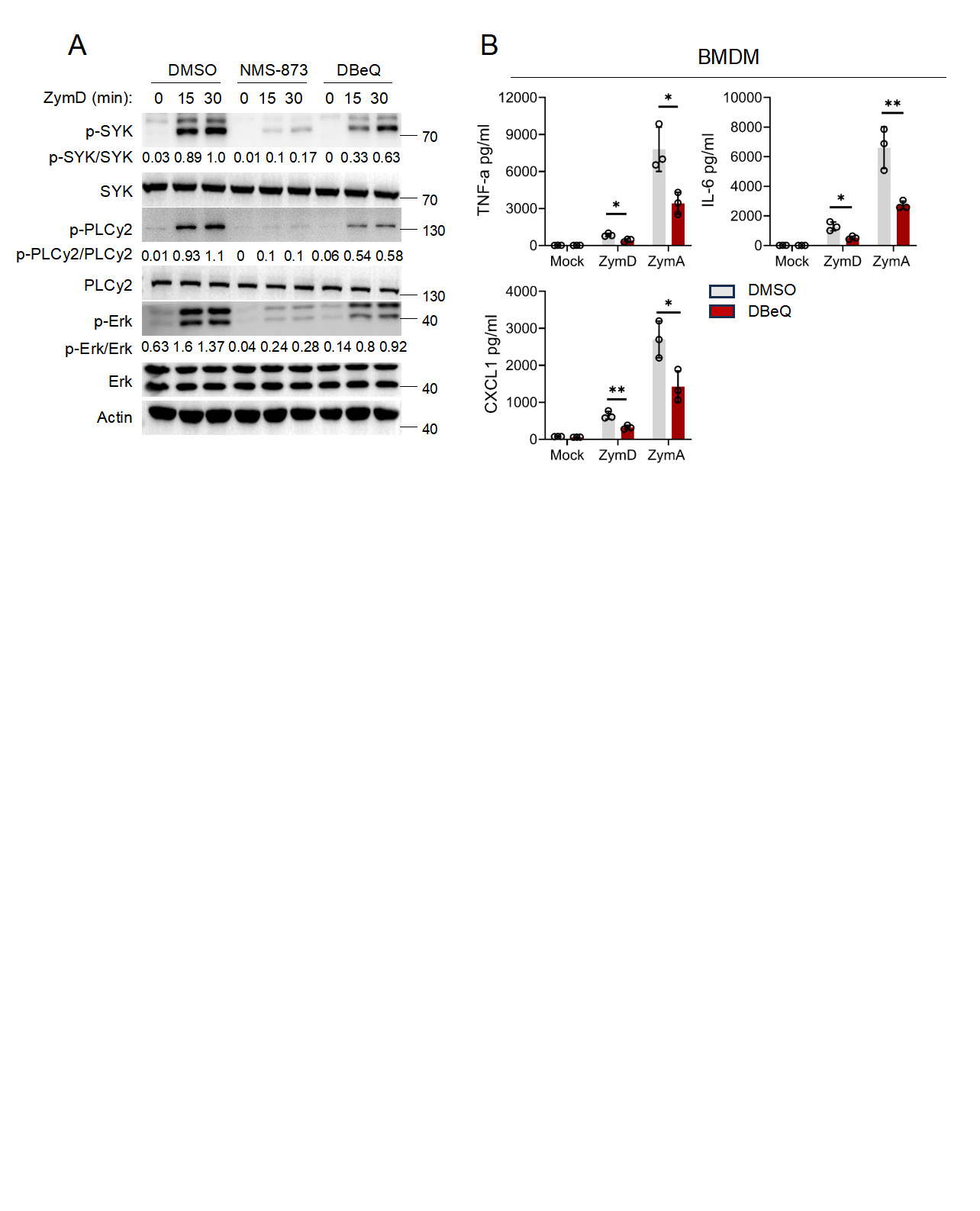

Supplement: S3 Fig — (A) Bone marrow-derived macrophages (BMDMs) from wild-type mice were pretreated with DMSO, NMS-873 (2 μM), or DBeQ (10 μM) for 1 hour, and then stimulated with Zymosan D (ZymD) (100 μg/ml) for the indicated times. This was followed by western blot analysis of the indicated proteins. (B) ELISA was performed for TNF-α, IL-6, and CXCL1 in BMDMs derived from wild-type mice, which were left unstimulated (Mock) or stimulated for 24 hours with ZymD (100 μg/ml) or Zymosan A (ZymA) (100 μg/ml), with DMSO or 2 μM of DBeQ. *: p<0.05; **: p<0.01; ***: p<0.001; ****: p<0.0001 based on two-tailed unpaired Student’s t test (B). (TIF) [file ppat.1012674.s003.tif]

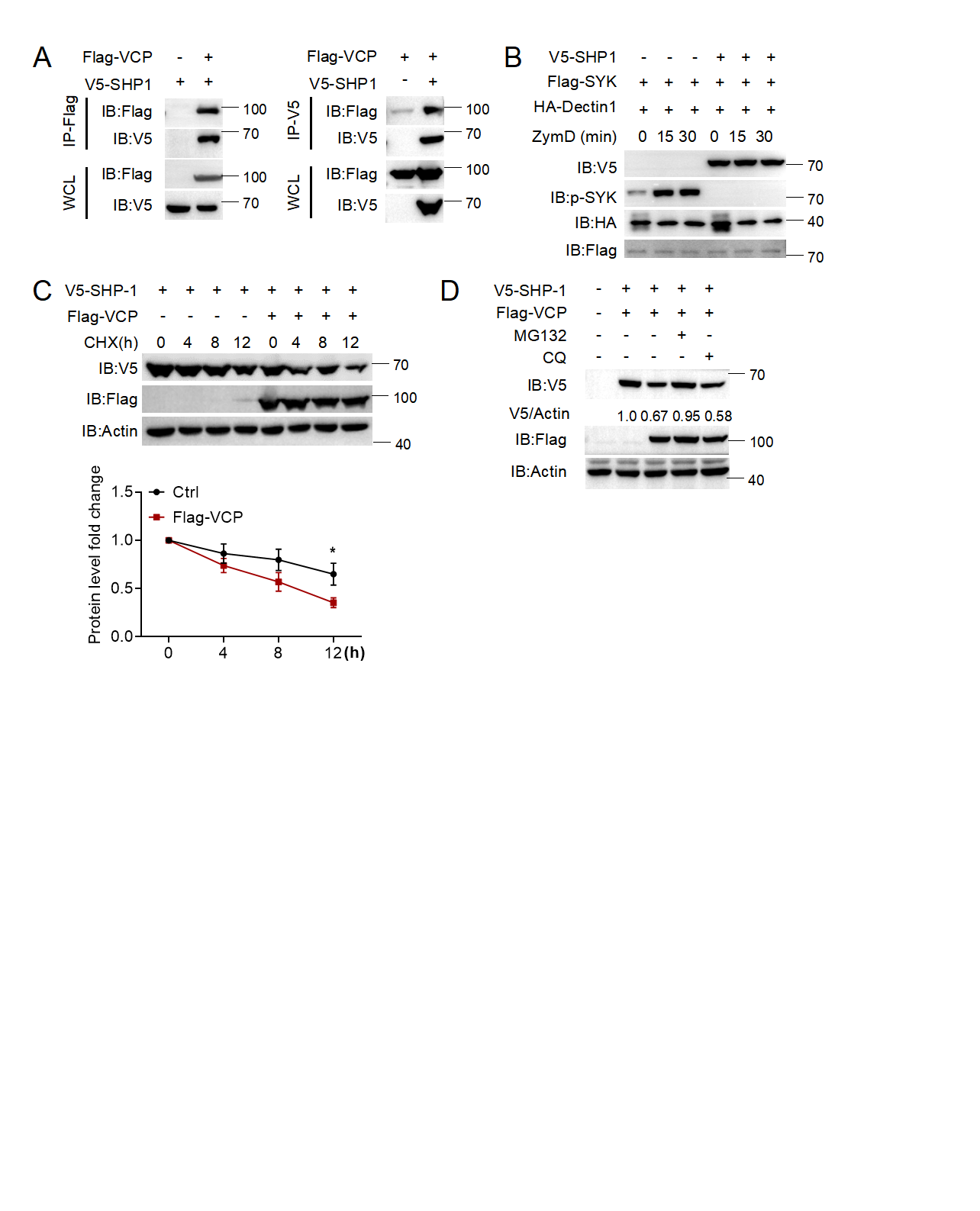

Supplement: S4 Fig — (A) Co-immunoprecipitation (Co-IP) analysis of the interaction between VCP and SHP1 in HEK293T cells transfected with Flag-VCP and V5-SHP1. (B) HEK293T cells were transfected with plasmids expressing SHP1, SYK, an empty control, or Dectin-1 for 36 hours and then stimulated with ZymD (100 μg/ml) for the indicated time. Cell lysates were immunoblotted with the indicated antibodies. (C) V5-SHP1 was transfected into HEK293T cells together with Flag-VCP and then treated with the protein synthesis inhibitor CHX for the indicated times. The protein levels of SHP1 were detected by immunoblot analysis. (D) V5-SHP1 was transfected into HEK293T cells together with Flag-VCP, followed by treatment with MG132 (10 μM) or chloroquine (CQ) (50 μM) for 10 hours. The protein levels of SHP1 were detected by immunoblot analysis. One representative experiment of three independent experiments is shown. (TIF) [file ppat.1012674.s004.tif]

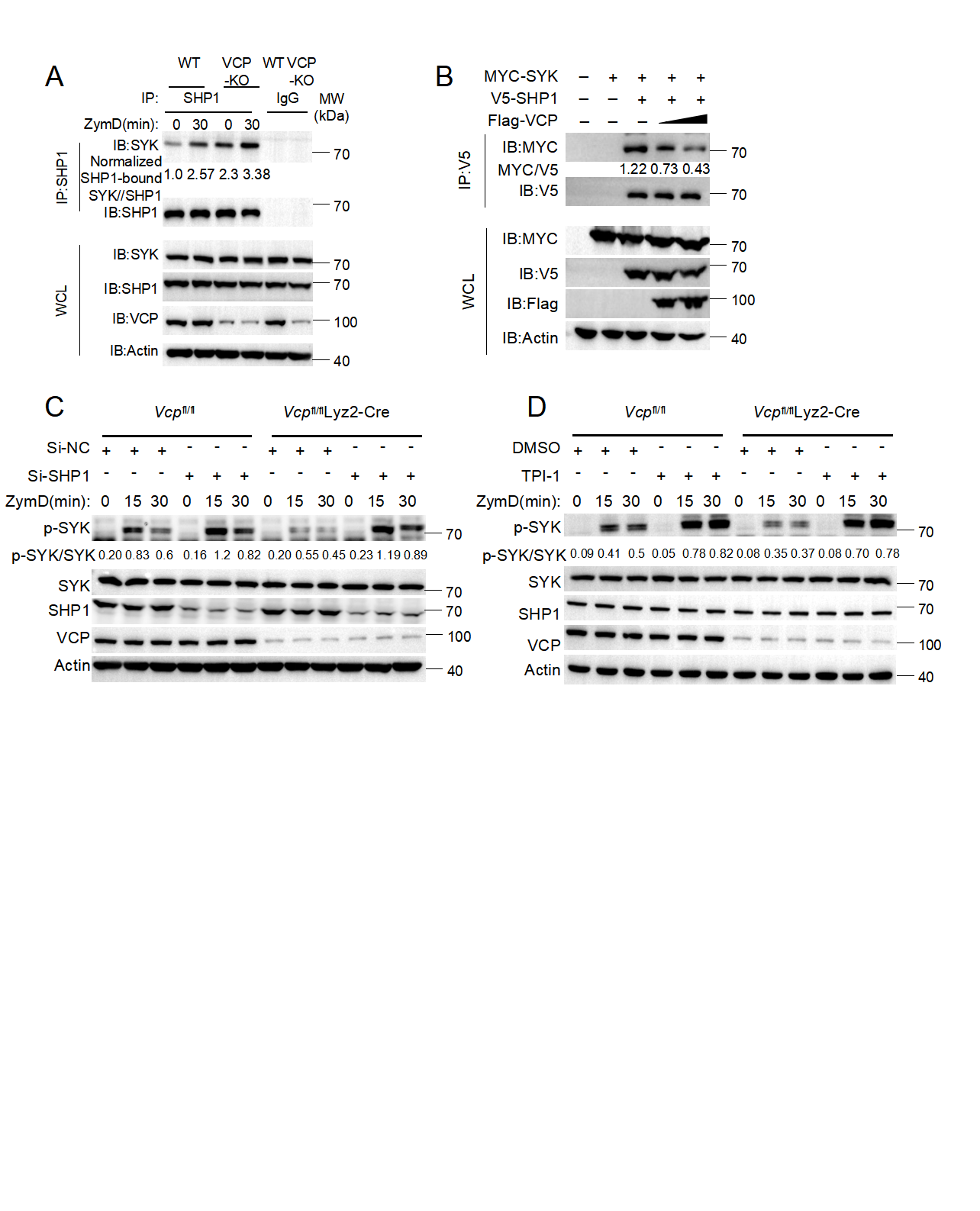

Supplement: S5 Fig — (A) Wild-type (WT) and VCP-deficient bone marrow-derived macrophages (BMDMs) were stimulated with ZymD (100 μg/ml) for the indicated times, and the cell lysates were subjected to immunoprecipitation (IP) with anti-SHP1 antibody (Ab), followed by Western blot analysis with anti-Syk Ab. (B) HEK293T cells were transfected with Myc-SYK, V5-SHP1, and Flag-VCP. They were then subjected to IP with anti-V5, and probed with the indicated antibodies (left margins). (C) WT and VCP-deficient BMDMs were transfected with si-NC or si-SHP1 for 5 days and then stimulated with ZymD for the indicated time points, followed by Western blot analysis of the indicated proteins. (D) WT and VCP-deficient BMDMs were pretreated with DMSO or the SHP1 inhibitor TPI-1 (10 μM) for 1 hour and then stimulated with ZymD for the indicated time points, followed by Western blot analysis of the indicated proteins. Data are representative of three independent experiments. (TIF) [file ppat.1012674.s005.tif]

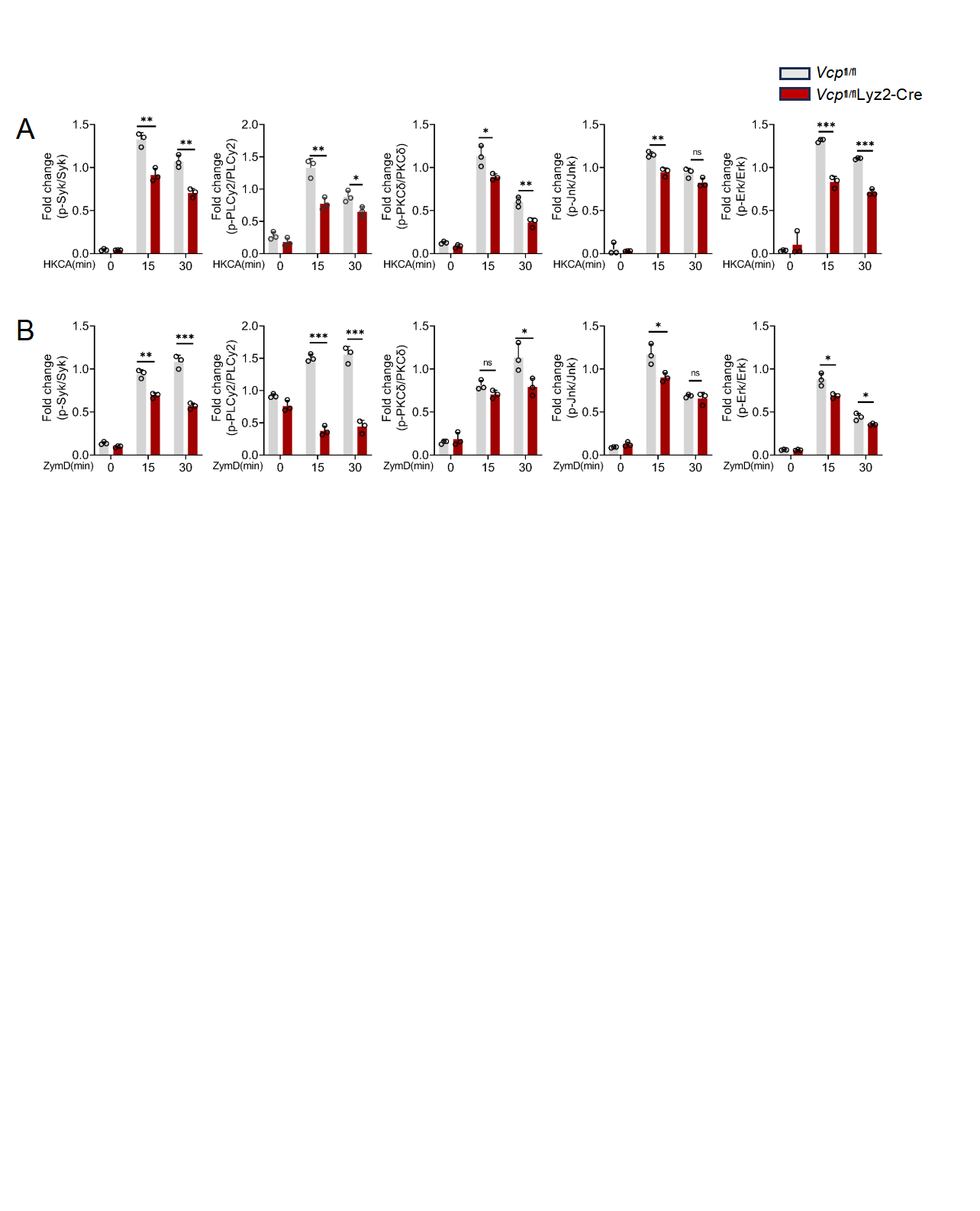

Supplement: S6 Fig — (A, B) Densitometric quantification of p-Syk, p-PLCγ2, p-PKCδ, p-JNK, and p-Erk, as shown in Fig 4A and 4B, was measured using ImageJ. The data are presented as mean ± SD and were analyzed using an unpaired two-tailed Student’s t-test. (A-B). (*: p<0.05; **: p<0.01; ***: p<0.001; ****: p<0.0001, ns: no significance) (TIF) [file ppat.1012674.s006.tif]

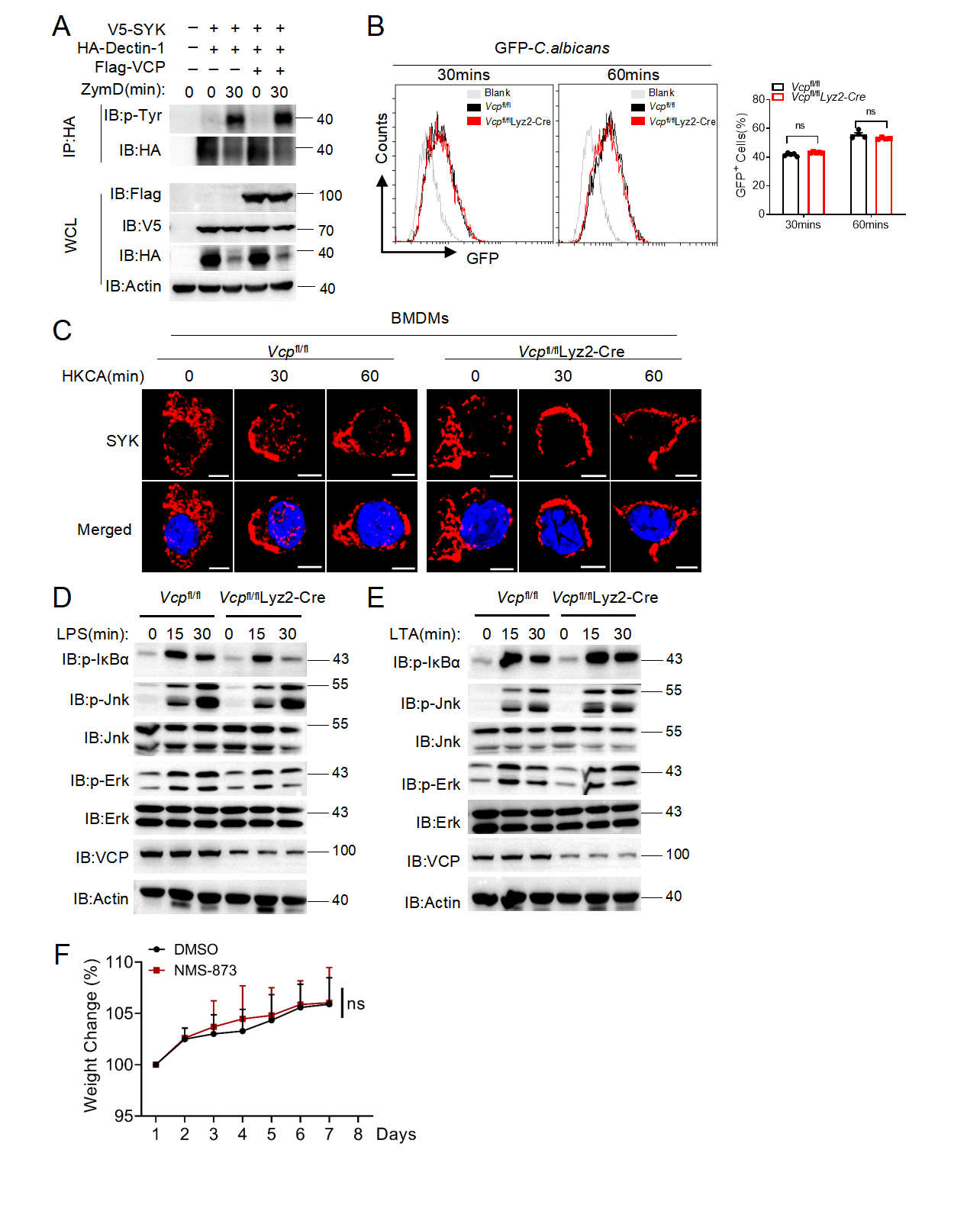

Supplement: S7 Fig — (A) HEK293T cells were transfected with plasmids expressing Dectin-1, SYK, an empty control, or VCP for 36 hours and then stimulated with ZymD (100 μg/ml) for the indicated times. Cell lysates were subjected to immunoprecipitation (IP) with anti-HA and then immunoblotted with the indicated antibodies. (B) Phagocytosis of Vcpfl/fl or Vcpfl/fl Lyz2-Cre BMDMs was evaluated by the method described in the Materials and Methods section. (C) Vcpfl/fl or Vcpfl/fl Lyz2-Cre cells were treated with HKCA (MOI = 1) for the indicated times, followed by immunofluorescence staining for the indicated proteins. Scale bar = 5 μm. (D-E) BMDMs from wild-type (WT) control mice or Vcp-deficient mice were stimulated with LPS (200 ng/ml) or LTA (100 μg/ml) for the indicated times, followed by Western blot analysis of the indicated proteins. (F) Six- to eight-week-old mice (5 pairs of sex- and age-matched littermates) were intraperitoneally (i.p.) injected with vehicle (DMSO) or NMS-873 (2 mg/kg) for three consecutive days, and their weight change was documented. Data are presented as mean ± SD and were analyzed using an unpaired two-tailed Student’s t-test (B), and two-way ANOVA (F) *: p<0.05; **: p<0.01; ***: p<0.001, ns: no significance. (TIF) [file ppat.1012674.s007.tif]
