# Supplementary material for: The protein segregase VCP/p97 promotes host antifungal defense via regulation of SYK activation
Source: PLoS Pathog. 2024 Oct 29;20(10):e1012674. doi: 10.1371/journal.ppat.1012674 (PMC11548748; doi:10.1371/journal.ppat.1012674)
Supplement: S1 Table — (DOCX) [file ppat.1012674.s008.docx]

**S1 Table. qPCR primers used in this study**

**qPCR primers**

| **Name** | **Purpose** | **Sequence(5'-3')** | **Source** |
| --- | --- | --- | --- |
| mouse Tnfa-F | For qPCR of Tnfa | GCCACCACGCTCTTCTGTCT | this study |
| mouse Tnfa-R | For qPCR of Tnfa | TGAGGGTCTGGGCCATAGAAC | this study |
| mouse Il6-F | For qPCR of Il6 | ACAACCACGGCCTTCCCTAC | this study |
| mouse Il6-R | For qPCR of Il6 | CATTTCCACGATTT CCCAGA | this study |
| mouse Il1b-F | For qPCR of Il1b | ACCTTCCAGGATGAGGACATGA | this study |
| mouse Il1b-R | For qPCR of Il1b | AACGTCACACA CCAGCAGGTTA | this study |
| mouse Il12b-F | For qPCR of Il12b | ATGTGGAATGGCGTCTC | this study |
| mouse Il12b-R | For qPCR of Il12b | GTCTCCTCGGCAGTT GG | this study |
| mouse Il23a-F | For qPCR of Il23a | GACTCAGCCAACTCCTCC | this study |
| mouse Il23a-R | For qPCR of Il23a | TGCTCCGTGGGCAAAGAC | this study |
| mouse Cxcl1-F | For qPCR of Cxcl1 | GCTCCTCCTTTCCAGGTCAGTT | this study |
| mouse Cxcl1-R | For qPCR of Cxcl1 | CGGTCAAAAA-GTTTGCCTTGAC | this study |
| mouse Cxcl2-F | For qPCR of Cxcl2 | CGGTCAAAAAGTTTGCCTTGAC | this study |
| mouse Cxcl2-R | For qPCR of Cxcl2 | GCCAAGGGTTGACTTCAAGAAC | this study |
| mouse Gapdh-F | For qPCR of Gapdh | TGGAGAAACCTGCCAAGTATGA | this study |
| mouse Gapdh-R | For qPCR of Gapdh | CTGTTGAAGTCGCAGGAGACAA | this study |
| Mouse β-actin-F | For qPCR of β-actin | CCACACCCGCCACCAGTTCG | this study |
| Mouse β-actin-R | For qPCR of β-actin | TACAGCCCGGGGAGCATCGT | this study |
