# Supplementary material for: The protein segregase VCP/p97 promotes host antifungal defense via regulation of SYK activation
Source: PLoS Pathog. 2024 Oct 29;20(10):e1012674. doi: 10.1371/journal.ppat.1012674 (PMC11548748; doi:10.1371/journal.ppat.1012674)
Supplement: S2 Table — (DOCX) [file ppat.1012674.s009.docx]

**S2 Table.** **The siRNA sequences of the oligos used in this study.**

**siRNA sequences**

| **Name** | **Sequence(5'-3')** |
| --- | --- |
| mouse VCP-1 sense | CCAUCCGUAAAGGAGAUAU |
| mouse VCP-1 antisense | AUAUCUCCUUUACGGAUGG |
| mouse VCP-2 sense | GGAGGAAUCCUUGAAUGAA |
| mouse VCP-2 antisense | UUCAUUCAAGGAUUCCUCC |
| mouse VCP-3 sense | GGAGCUGAUUUGACAGAAAUU |
| mouse VCP-3 antisense | UUUCUGUCAAAUCAGCUCCAG |
| mouse SHP1(Ptpn6)-1 sense | GAGCAAGAAGGAAGAGAAATT |
| mouse SHP1(Ptpn6)-1 antisense | UUUCUCUUCCUUCUUGCUCTT |
| mouse SHP1(Ptpn6)-2 sense | AGAAGAAACUGGAGAUCAUTT |
| mouse SHP1(Ptpn6)-2 antisense | AUGAUCUCCAGUUUCUUCUTT |
| mouse SHP1(Ptpn6)-3 sense | UGACAGAGCUGGUCGAGUATT |
| mouse SHP1(Ptpn6)-3 antisense | UACUCGACCAGCUCUGUCATT |
| Negative control sense | UUCUCCGAACGUGUCACGUTT |
| Negative control antisense | ACGUGACACGUUCGGAGAATT |
| Mouse GAPDH sense | UUGAUGACAAGCUUCCCAUUCUTT |
| Mouse GAPDH antisense | AGAAUGGGAAGCUUGUCAUCAATT |
