# Supplementary material for: The protein segregase VCP/p97 promotes host antifungal defense via regulation of SYK activation
Source: PLoS Pathog. 2024 Oct 29;20(10):e1012674. doi: 10.1371/journal.ppat.1012674 (PMC11548748; doi:10.1371/journal.ppat.1012674)
Supplement: Attachment — Submitted filename: 4 point-by-point response.docx [file ppat.1012674.s012.docx]

Point-by-point response to the reviewers

Part I –Summary Please use this section to discuss strengths/weaknesses of study, novelty/significance, general execution and scholarship.

Reviewer #1: This manuscript describes a role for the ATPase VCP in promoting antifungal innate immunity. Specifically, the authors demonstrate that the ATPase activity of VCP promotes signaling downstream of Dectin-1 to support cytokine production in response to Dectin-1 ligands or the fungal pathogen *Candida albicans*. They propose a model in which VCP and its co-factor UFD1 pull ubiquitinated SHP-1 out of the SYK complex during Dectin-1 stimulation, thus limiting the negative regulatory effect of SHP-1 on SYK activation. Importantly, VCP deletion in neutrophils and macrophages, or VCP inhibition in vivo rendered mice susceptible to system *C. albicans* infection. While this study clearly supports a role for VCP in promoting Dectin-1/SYK signaling, many parts of the model are not directly tested, or only weakly supported. Additionally, the results would be more interpretable with increased rigor and more transparent data presentation.

We truly appreciated the positive comments from Reviewer#1, and have added substantial amounts of data to address this reviewer’s concerns as described below.

Reviewer #2: C-type lectin receptors (CLRs, e.g., Dectin-1) play a critical role in host recognition of fungal PAMP to mount host defense against fungal infections, including *Candida infections*. In this report, authors identified a new regulator in the Dectin-1/SYK/SHP-1 signaling cascade, the ATPase valosin-containing protein (VCP)/p97 and its co-factor UFD1. Their data showed that VCP promotes the ubiquitination and degradation of SHP-1, a negative regulator of SYK function, to activate SYK and positively regulate Dectin-1 signaling. Their model suggests that VCP may help pull SHP-1 out of the SYK complex for degradation. Such dissociation activates SYK mediated downstream signal to induce pro-inflammatory cytokine production. As expected, the VCP defective animals showed increased susceptibility against *Candida* infection. Overall, this is an interesting study with some novel findings in the Dectin-1 signal pathway regulation. Some of the data, however, are not very convincing and may need additional verification.

We are grateful for the support and constructive suggestions from Reviewer#2. We have now provided a number of new data to consolidate the conclusions as elaborated in the following.

Reviewer #3: Shao et al explored the roles of the VCP segregase in the Dectin-1 signaling in recognition of the fungal pathogen *C. albicans*. Dectin-1 is a major PRR sensing beta-glucan in the fungal cell wall and therefore plays essential roles in host defense against pathogenic fungi. In this study, the authors found that VCP participates in this dectin-1 recognition pathway, especially via SYK, in response to *C. albicans*. This manuscript would be of highly interest if the data presented support the conclusion. Many of the western blot data claiming that VCP is phosphorylated upon dectin-1 signal, which is involved in SYK phosphorylation are not convincing. ‘Significant difference’ cannot be stated without a statistic verification, which is lacking in this manuscript.

In addition, the attenuation of cytokine productions in VCP knock-down mutants or with VCP inhibitors appeared to be partial, which means the conclusion or the title is too strong. This manuscript therefore requires substantial amount of revisions to support the conclusion.

We are thankful for reviewer#3’s insightful suggestions. We have conducted a large number of experiments to strengthen our conclusions, and performed statistical analysis thoroughly to improve data presentation.

Also, we have quantified the Western blot data, which are shown in the new Figure S2 and Figure S6.

We also agree with this reviewer that the effect of VCP knock-down or inhibition on cytokine production is partial, thus have modified the conclusion accordingly (lines 205, 235 and 236).

--------------------

Part II –Major Issues: Key Experiments Required for Acceptance.

Reviewer #1: Major Comments:

1) In all cases where ELISA or RT-qPCR data are present, the authors show technical replicates from a single experiment, rather than biological replicates from 3 experiments. These technical replicates are not appropriate for statistical analyses. Biological replicate data should be shown. Mean scaling or replicate matching can be performed if the strength of responses is variable between experiments, but if the differences are not significant across biological replicates, this would greatly diminish the strength of their conclusions.

We are grateful for the reviewer’s suggestion! Now we have pooled ELISA and RT-qPCR data from three independent experiments in the revised manuscript, and stated them as “biological replications” in the figure legends of the revised manuscript (Figure 1G, H, Figure 2C, D, Figure 4C, D and Figure S3B).

2) In addition to performing statistical testing on biological replicates rather than statistical replicates, the authors should perform appropriate statistical tests, such as ANOVA, rather than t-tests.

This point is well-taken! We have now conducted statistical analysis thoroughly. Figure 1G, Figure S2A and S2B were analyzed by one-way ANOVA. Figure 5B, 5F and Figure S7F were analyzed by two-way ANOVA. Log-rank test was performed for Figures 5A and 5E.

3)	The model proposed by the authors is logical given the known role of VCP, but it is not directly experimentally tested. The authors should perform additional experiments to show that VCP is indeed pulling SHP-1 out of the complex and that VCP affects SHP-1 protein stability directly. For example, is the protein half-life of SHP-1 affected by VCP expression perturbations? Does proteasome inhibition restore SHP-1 protein levels in VCP knockdown or overexpressing cells? This assumption needs to be experimentally validated before drawing these conclusions.

Per the reviewer’s suggestion, we have performed additional experiments to support the scenario that VCP is indeed pulling SHP-1 out of the SYK complex! Please find the new data in Figures 3I-J and S5A-B. Also, we have provided new data showing that overexpressed VCP accelerated the degradation of SHP1 and proteasome inhibition MG132 could restore SHP-1 protein levels in VCP overexpressing cells. Please find the new data in the Figures S4C, S4D of the revised manuscript.

4)	The authors describe "screening a panel of antibodies recognizing proteins with MWs of ~100 kDa and found VCP was enriched in anti-P-Tyr Ips during dZymo treatment. What proteins did they screen? Why focus on 100 kDa? The logic of this is not clear.

We screened the proteins about 100 kDa by mass spectrometry, which is now included in new Figure 1B revealing VCP as one of the p-Tyr proteins enriched.

To be specific, ZymD stimulation induced a myriad of tyrosine phosphorylation modifications on various proteins, primarily on proteins of 70-85, 100 or 125 kDa. Since the tyrosine-phosphorylation of SHP2 (~72 kDa), SYK (~72 kDa), PKC-δ (~78kDa), EPHB2 (~130kDa) and PLCγ2 (~150kDa) have already been reported to be critical in antifungal pathways, we focused on 100 kDa proteins in the pursuit of new tyrosine-phosphorylated proteins in fungal infection.

5)	After comparing multiple inhibitors that block VCP ATPase activity, the authors go forward with only NMS-873, which has the strongest effect. It even appears that the other inhibitors have no effect. This could potentially select for an inhibitor with off-target effects—this has not been ruled out by the authors, and thus diminishes the strength of the conclusions based on this inhibitor activity.

To rule out the non-specific effect of VCP inhibitor NMS-873, we tested another inhibitor of VCP, and found that DBeQ showed a similar effect to NMS-873. Please find the new data in Figures S3A, S3B of the revised manuscript.

6)	A causal effect of SHP-1 removal by VCP and its effect on SYK signaling is not demonstrated. Does VCP still affect SYK activation in SHP-1 knockout cells?

We have now validated the role of VCP in regulating SYK signaling! Upon using siRNA to knockdown SHP-1 or the inhibitors of SHP-1 (TPI) in VCP knockout BMDM cells, we found that VCP didn’t affect SYK activation when SHP-1 was knockdown or its enzyme activity was suppressed, hence supporting our hypothesis that VCP promotes SYK activation through degrading SHP-1. Please find the new data in Figures S5C, S5D of the revised manuscript.

7)	It is not tested whether the effect of VCP on signaling could be upstream of SYK, such as Dectin-1 activation or defective phagocytosis. The authors should directly test these to test whether these alternate models would explain their data.

We are thankful for the reviewer’s suggestion, and have performed additional experiments accordingly. Our new results indicate that VCP does not have an impact on Dectin-1 phosphorylation or phagocytosis of fungi, which are included in the new Figures S7A, S7B of the revised manuscript.

8)	Many IP experiments are difficult to interpret because IgG pulldown has nearly the exact same banding pattern (albeit weaker) than the target pulldown.

We have now conducted those IP experiments with IgG pulldown as a control in the revised manuscript, and the new data are included in Figures 3A,3D,3E.

Reviewer #2: I have the following specific comments.

1. VCP is known to be involved in ubiquitin-dependent processes and regulates multiple cellular functions. It is not clear whether the VCP regulation of Dectin-1/SYK signaling is specific or just a moonlight activity? A brief discussion would be helpful.

We want to thank reviewer#2 for his/her insightful suggestions! We have now used lipopolysaccharide (LPS) and lipoteichoic acid (LTA) to stimulate the VCP-WT and KO macrophages, and found that VCP-deficiency didn’t impact on TLR4 or TLR2 signaling pathways. Therefore, VCP regulation of Dectin-1/SYK signaling appear to be specific. Please find the new data in Figures S7D, S7E of the revised manuscript.

MYO1F has also been reported to interact with the SYK to translocate it from membrane to cytosol. Whether is necessary to include this protein in the model?

Per this reviewer’s suggestion, we conducted the immunofluorescence experiment to test whether VCP regulates SYK translocation. The result indicated that VCP-deficiency did not affect the translocation of SYK from the cytosol to the cell membrane. Please find the new data in Figure S7C of the revised manuscript.

2.	The study mainly drew conclusion based on western blot data. While a large amount work is presented, some data are not as clear cut as one would expect. For those protein signals, a quantification of image signal would be helpful.

This point is well-taken! We have now quantified the key protein signals, which are included in Figure S2 and Figure S6 of the revised manuscript.

Figure 1A and 1B showed the proteins phosphorylated by Tyrosine kinase. It may be necessary to label clearly what are the bands described in the text (based on MW).

We have now labelled the band by a red arrow (Figure 1A).

If those differently presented bands in ZymD treated samples are the targets of interest, why phosphorylation leads to such a significant change of the MW? Also Figure 1A may need a loading control. For some of those important western blot results, the original images may be attached as supplement material for review.

The MW changes observed could be due to other modifications, such as ubiquitination/sumoylation also occurred along with tyrosine phosphorylation. Alternatively, multiple ser/Thr- and tyr-phosphorylation events could be simultaneously induced by ZymD stimulation. Although we don’t have a definitive answer for this intriguing observation at the moment, we have now attached the original images as supplemental material for this reviewer’s evaluation.

3.	Albeit weaker signals, the Si-VCP treatment in figure 1E does not seems to cause significant gene silencing based on the p-SYK protein signal. In the text, they stated that such a modest reduction of protein expression/phosphorylation triggered a significant difference in pro-inflammatory cytokine production (line 157), but the Figure 1F does not reflect that. Both 1F and 1G seems to show the result of VCP overexpression data. There are quite a few places with wrong information, which makes reading this ms a bit challenging.

We sincerely apologize for this inadvertent mistake! We have now corrected it and presented this data in Figure 1G in revised manuscript.

Reviewer #3: (No Response)

--------------------

Part III – Minor Issues: Editorial and Data Presentation Modifications.

Reviewer #1: Minor Comments:

1)	For major findings, the authors should consider quantification of western blots, which could be added to the supplemental materials.

We are thankful for the reviewer’s suggestion, and have quantified the respective protein of western blots. Please find the data in the Figure S2 and Figure S6 of the revised manuscript.

2)	There are several examples of figure call-outs in the text not matching the figures. Examples: Figure 1F – text says siRNA knockdown of VCP leads to less cytokine production, but the graphs show more cytokine production following VCP overexpression.

We are thankful for the reviewer’s suggestion, and apologize for such a mismatch. We have complemented this data in revised manuscript (Figure 1G).

In Figure 3A, the text says that VCP interacts with SHP-2, but this is not shown in the immunoblot. Figure 3F is mis-called in the text as Figure 3G. Figure 5A and B are swapped in the text call-out.

We are thankful for the reviewer’s suggestion! We are so sorry for mistaking the writing. This place is “Dectin-1”, not “SHP-2”, and we have changed the error. We also changed “Figure 3G” to “Figure 3F” in revised manuscript, and Figure 5A and 5B were reversing in revised manuscript.

3)	The text for Figure 5F claims that NMS treatment in infected mice led to more weight loss, but there are no statistics for this analysis and it does not appear to be statistically significant.

We are thankful for the reviewer’s suggestion, and performed the statistical differences of the data by two-way ANOVA and the *P* value was 0.028. Please find the data in the Figure 5F of the revised manuscript.

4)	The authors note that NMS treatment did not affect the overall health of mice in the absence of infection, but these data are not shown. This needs to be included.

We are thankful for the reviewer’s suggestion, and have added these data. Please find the data in Figure S7F of the revised manuscript.

Reviewer #2:

4. Why use ZymD and ZymA as Dectin-1 signal inducer for different experiments. A brief explanation or comparison may be necessary. Also the data on p-SYK in figure 2A and 2B showed ZymA has a much stronger induction, while a much stronger inhibition is observed following 5 µM NMS-875 treatment. Some discussion about these two ligands may be needed.

We are deeply appreciative of the reviewer's insightful comments, thus have used both ZymD and ZymA as inducers of Dectin-1 signaling in our experiments. ZymA, a preparation from the fungal cell wall, contains ligands for both Dectin-1 and TLR2, hence capable of eliciting a more pronounced signaling response compared to ZymD which only engages Dectin-1 (PMID: 12719479). On the other hand, ZymD is devoid of TLR2 agonists, thus acting as a specific Dectin-1 ligand and allowing the precise examination of the contribution of VCP to Dectin-1 signaling. This explanation has been included in the revised manuscript lines 212, 213 and 214.

5.	Figure 2E, the VCPK542A mutant can still partially recover the p-SYK signal following ZymD stimulation. Does that mean the ATPase activity is required but not essential?

We agreed with the reviewer's assertion that VCP’s ATPase activity may partly contribute to VCP’s impact on SYK activation.

6.	Figure 3A, seems like without ZymD, VCP can also interact with Dectin and SYK.

We are thankful for the reviewer’s careful observation. The data in Figure 3A indeed suggests VCP could also interact with Dectin and SYK without ZymD stimulation, but we are not sure about its physiological significance because the interaction was very weak.

Why has the MW (KDa) increase shown in the figure 3G and 3H? What does it indicate?

It is possible that the increased MW of SHP-1 is a result of the overexpression of VCP or UFD1 in HEK293 cells promoting the ubiquitination and degradation of SHP-1. We also confirmed that inhibition of proteasome degradation pathway by MG132, which could reverse the degradation of SHP1 with VCP overexpression (Figure S4D). Please look into lines 271-275 of the manuscript for details.

If authors want to show an increased ubiquitination of SHP-1, the polyubiquitin signal is quite weak, thus not very convincing. No relevant explanation was given.

We are thankful for the reviewer’s question! We have repeated this experiment and detected strong polyubiquitination of SHP-1. Please find the new data in Figure 3F of the revised manuscript.

Also Figure 3F legend does not make sense. Please double check all figure legends for accuracy.

We are thankful for the reviewer’s suggestion! We have now corrected the figure legend for Figure 3F and we had double checked all figure legends including Figure 3F legend.

7.	Figure S1. No figure legend. Also why there are two panels of IB:p-SYK in the figure?

We apologize for such an error, and have added the Figure legend to Figure S1. Two panels in the immunoblot (IB) for p-SYK represent the bands from long- and short-time exposures, respectively. We have also removed the panel showing the long exposure of p-SYK from the revised manuscript (Figure S4B).

Reviewer #3: (No Response)

--------------------

Overall, we are truly grateful for the insightful comments of three reviewers, and sincerely hope this manuscript will be acceptable for the journal of PLOS Pathogens.
