# Supplementary material for: The protein segregase VCP/p97 promotes host antifungal defense via regulation of SYK activation
Source: PLoS Pathog. 2024 Oct 29;20(10):e1012674. doi: 10.1371/journal.ppat.1012674 (PMC11548748; doi:10.1371/journal.ppat.1012674)
Supplement: Attachment — Submitted filename: point-by-point response.docx [file ppat.1012674.s013.docx]

Point-by-point response

We are thankful for both editor and reviewers’ comments and suggestions, which helped us to further improve the quality of this manuscript. Please find our point-by-point response below:

Editor Comments:

Thank you very much for submitting your manuscript "The protein segregase VCP/p97 promotes host antifungal defense via regulation of SYK activation" for consideration at PLOS Pathogens. As with all papers reviewed by the journal, your manuscript was reviewed by members of the editorial board and by several independent reviewers. The reviewers appreciated the attention to an important topic. Based on the reviews, we are likely to accept this manuscript for publication, providing that you modify the manuscript according to the review recommendations.

We are thankful for the editor’s comments, and we performed the modifications suggested by the reviewers to address the reviewers’ concerns.

The authors addressed most of previous reviewers' editorial and experiment comments in this revised manuscript. The reviewers also agreed that the revised manuscript is much improved. Having said that, they still raised some concerns that need to be addressed in the second round of revision, as can be seen in the reviewers' comments. These include more quantitative measurement of VCP phosphorylation (reviewer 1),

We are thankful for the reviewer’s suggestion, and we performed another three experiments of VCP phosphorylation and quantified the western blot data, and please find the statistical data in the Figure 1D of the revised manuscript and the raw data below:

providing raw blotting data for Ig pulldown (reviewer 3), and several comments from reviewer 2. I think that the authors could reasonably address these questions.

We are grateful for the editor's suggestion. In response, we have provided the raw blotting data for the Ig pulldown in the Supplemental Information section and have provided a detailed response to Reviewer 2's comments.

Reviewer's Responses to Questions

**Part I - Summary**
Please use this section to discuss strengths/weaknesses of study, novelty/significance, general execution and scholarship.

Reviewer #1: This is a revised manuscript and the authors responded to reviewers' point by providing additional experiments and adding more robust statistic analysis. Overall responses are acceptable. However, the phosphorylation of VCP upon dectin-1 signaling is not really convincing based on western blot experiment in fig 1. What's difference between no activation (0 zyme) vs activation (Zymo treatment)? They need to provide clear, convincing evidence to make the case.

We are thankful for the reviewer’s suggestion, and we performed another three experiments of VCP phosphorylation and quantified the western blot data, and please find the statistical data in the Figure 1D of the revised manuscript and the raw data below:

Reviewer #2: The revised submission has addressed most of my concerns and the quality has been improved. Impressive amount of work has been included. I have a few additional comments.

1. It is nice to have the ATPase activity-dead VCP mutant. Not sure whether such mouse mutant strain is available. To confirm the ATPase activity of VCP is required for fungal infectivity, in addition to using the NMS-872 inhibitor, testing the VCP (K542A) ATPase-dead mutant mouse strain would be more convincing.

We appreciate the reviewer's suggestion. We have found a VCP^K524A^ transgenic mouse model from Jackson Lab, which overexpresses a K524A (lysine to alanine) point mutant form of mouse Vcp cDNA under the control of the mouse cardiac-specific Myh6 and tetO promoters (PMID:16234241). However, a VCP^K524A^ transgenic mouse model with specific expression of the K524A point mutant form in immune cells or the whole body has not been developed.

What is the rationale to use NMS-872 at 2 mg/Kg dosage?

We are thankful for the reviewer’s suggestion, and we performed pre-experiments to explore what dosage of NMS-873 was suitable for mice. We found that 2 mg/Kg dosage of NMS-873 was the optimal concentration to induce mouse death based on the weight and survival curve, and please find the data below:

2. Line 401, please cite a reference on the role of VCP as an ATPase in targeting and transporting ubiquitinated substrate for degradation.

We are thankful for the reviewer’s suggestion, and we added the reference in the revised manuscript and please find the content in the line 402 of the revised manuscript.

3. Figure 2B. The image used for the ikBa signals is partially cut out and should use a better image with complete bands. Also comparing with the original blot (have two strong bands), the up band was cut out. Please explain what is the up band and why was it cut out?

We are thankful for the reviewer’s suggestion, and we considered the up band of IκBα might be p-IκBα or a non-specific band. In order to conform the result, we re-tested the total protein of IκBα and p-IκBα, and the image was showed below. We also replaced the image by a better image and please find the data in the Figure 2B of the revised manuscript.

4. Figure 3E. please add the label to the right panel.

We are thankful for the reviewer’s suggestion, and we added the right panel in the revised manuscript and please find the data in the Figure 3E of the revised manuscript.

5. Figure 6. In the model, the letters on the dectin-1 phosphorylation site are hard to see. May change the color of the letters.

We are thankful for the reviewer’s suggestion, and we re-marked the dectin-1 phosphorylation site with yellow font and please find the Figure 6 of the revised manuscript.

Reviewer #3: The authors’ revisions, including toning down their language regarding the role of VCP in regulation of Dectin-1 signaling, quantification of experimental replicates, and new data have strongly improved the manuscript. This is an important study identifying a new regulator of CLR signaling. The authors have addressed our concerns well and their data now fit their conclusions.

We are deeply appreciative of the reviewer's insightful comments.

**Part II – Major Issues: Key Experiments Required for Acceptance**
Please use this section to detail the key new experiments or modifications of existing experiments that should be absolutely required to validate study conclusions.

Generally, there should be no more than 3 such required experiments or major modifications for a "Major Revision" recommendation. If more than 3 experiments are necessary to validate the study conclusions, then you are encouraged to recommend "Reject".

Reviewer #1: Further verification of phosphorylaion of VCP upon dectin-1 activation is needed.

We are thankful for the reviewer’s suggestion, and we performed another three experiments of VCP phosphorylation and quantified the western blot data, and please find the statistical data in the Figure 1D of the revised manuscript and the raw data below:

Reviewer #2: (No Response)

Reviewer #3: (No Response)

**Part III – Minor Issues: Editorial and Data Presentation Modifications**
Please use this section for editorial suggestions as well as relatively minor modifications of existing data that would enhance clarity.

Reviewer #1: (No Response)

Reviewer #2: (No Response)

Reviewer #3: (No Response)

PLOS authors have the option to publish the peer review history of their article ([what does this mean?](https://c05y1x9s.r.us-east-2.awstrack.me/L0/https:%2F%2Fjournals.plos.org%2Fplospathogens%2Fs%2Feditorial-and-peer-review-process%23loc-peer-review-history/1/010f0191d9332cfc-be5b73ab-5e53-4766-bc35-895ffb1294f4-000000/BamelttRa7nDinErBkwMogRA4sY=175)). If published, this will include your full peer review and any attached files.


If you choose “no”, your identity will remain anonymous but your review may still be made public.


**Do you want your identity to be public for this peer review?** For information about this choice, including consent withdrawal, please see our [Privacy Policy](https://c05y1x9s.r.us-east-2.awstrack.me/L0/https:%2F%2Fwww.plos.org%2Fprivacy-policy/1/010f0191d9332cfc-be5b73ab-5e53-4766-bc35-895ffb1294f4-000000/NnUNmHDqYwBNK5UPe9Cjb-yUXvU=175).

Reviewer #1: No

Reviewer #2: No

Reviewer #3: No

Overall, we extend our heartfelt thanks to both the reviewers and the editor for their insightful comments and constructive suggestions. Their feedback has been invaluable in enhancing the quality of our study.
